# Composites Based on Collagen, Chondroitin Sulfate, and Sage Oil with Potential Use in Dentistry

**DOI:** 10.3390/biomimetics11010008

**Published:** 2025-12-24

**Authors:** Bogdan Valeriu Sorca, Ana-Maria Rosca, Durmuş Alpaslan Kaya, Sergiu-Marian Vatamanu, Mădălina Georgiana Albu Kaya, Cristina Elena Dinu-Pîrvu, Mihaela Violeta Ghica, Alina Elena Coman, Laura Cristina Rusu, Irina Titorencu

**Affiliations:** 1 Department of Oral Pathology, Multidisciplinary Center for Research, Evaluation, Diagnosis and Therapies in Oral Medicine, “Victor Babes” University of Medicine and Pharmacy Timisoara, 2 Eftimie Murgu Sq., 300041 Timisoara, Romania; bogdan.sorca@umft.ro (B.V.S.); laura.rusu@umft.ro (L.C.R.); 2 Institute of Cellular Biology and Pathology “Nicolae Simionescu”, 8 B. P. Hasdeu Street, District 5, 050568 Bucharest, Romania; ana-maria.rosca@icbp.ro (A.-M.R.); marian.vatamanu@icbp.ro (S.-M.V.); 3 Department of Field Crops, Faculty of Agriculture, Hatay Mustafa Kemal University, Antakya-Hatay 31034, Turkey; dkaya@mku.edu.tr; 4 Collagen Department, INCDTP—Division Leather and Footwear Research Institute, 93 Ion Minulescu Str., 031215 Bucharest, Romania; madalina.albu@icpi.ro (M.G.A.K.); alina.coman@icpi.ro (A.E.C.); 5 Department of Physical and Colloidal Chemistry, Faculty of Pharmacy, Carol Davila University of Medicine and Pharmacy, 6 Traian Vuia Str., 020956 Bucharest, Romania; cristina.dinu@umfcd.ro (C.E.D.-P.); mihaela.ghica@umfcd.ro (M.V.G.); 6 Innovative Therapeutic Structures Research and Development Center (InnoTher), Carol Davila University of Medicine and Pharmacy, 6 Traian Vuia Str., 020956 Bucharest, Romania

**Keywords:** bone tissue regeneration, cell biocompatibility, collagen–chondroitin–sage composite, sponge form

## Abstract

Osseointegration in dental implants involves the use of materials that mimic the bone tissue, with special properties such as biocompatibility and biodegradability. In this study, we describe the preparation and characterization of composites based on collagen, chondroitin sulfate, and sage oil obtained by freeze-drying method. Their morphological structures were determined by water uptake and scanning electron microscopy, the physical–chemical interactions between components by FT-IR, the stability by in vitro collagenase degradation, and the results indicate that the samples’ properties are highly influenced by the hydrophobic and hydrophilic character of sage essential oil and chondroitin sulfate, respectively, concluding that we can design a formulation with certain properties. The composite spongious forms were evaluated for cytocompatibility using the MG63 osteoblast cell line and subjected to histological observation. The results showed that the samples with sage essential oil were most resistant to enzymatic degradation, and the ones with chondroitin sulfate promoted the deposition of an abundant extracellular matrix. Taken together, the results suggest that incorporating chondroitin sulfate and sage oil in a controlled manner into collagen scaffolds represents a promising approach for enhancing bone tissue regeneration.

## 1. Introduction

The market size of the worldwide dental implants is continuously growing, with an estimated value of USD 10.4 billion in 2023. Because of the limitations of removable prosthetics, discomfort, lack of natural appearance, and need for maintenance, the acceptance level for dental implants is increasing among patients [1], accounting for about 3.5 billion people all over the world suffering from oral diseases [2].

Osseointegration in dental implants involves the connection between living bone and the surface of the implant. This process can take several months [3,4], starting with the placement of the implant, which is considered by the body as a foreign object, continuing with new bone formation by the osteoblasts, and ending when the implant becomes an integral part of the jawbone. During the process of osseointegration, the biocompatibility of the osteoblasts with the implant is crucial in successful implantation because the cells are responsible for accepting or rejecting the new implant [5]. The failure of implants does not happen very often, but the osseointegration can receive early or late responses to the implanted biomaterials [6]. In order to enhance osseointegration time and quality, the implant can be coated with some bioactive materials with osteoinductive properties, or additional materials as a scaffold for bone growth can be a good choice during the implantation process [7]. Implant coatings, capable of actively interacting with the surrounding tissues, are important in order to promote dental implant acceptance and rapid recovery.

The importance of type I collagen (COLL)—the main component of the extracellular matrix—in the body and its wide applications in dentistry and medicine is well known [8,9]. Collagen has an active role in osseointegration of dental implants, helping to enhance the bone regeneration, improving bone tissue adhesion to the implant, and facilitating the healing zone [10].

The literature mentions various composites meant for dentistry purposes, in order to help surgeons and patients. In addition to collagen, chondroitin sulfate (CS) is intensively used in the coating of dental implants. CS is a natural glycosaminoglycan, part of the cartilage and bone structure, aiming at bone regeneration and tissue elasticity [11,12]. It increases the activity of osteoblasts and has a favorable effect on bone formation, promoting faster and more stable implant integration. It also has anti-inflammatory effects, which can reduce postoperative swelling and discomfort [13]. Schneiders et al. presented in their studies on small animals that the addition of CS to composites based on collagen and hydroxyapatite improves bone remodeling from the beginning of bone healing [14].

The interaction between COLL and CS was studied. To modify the COLL scaffold, CS was attached. Changes in COLL were observed at microscopic level during this modification. The results suggested that CS could have a good influence on COLL, improving the biocompatibility and mechanical properties, among others [15].

Another animal study, which evaluated the osseointegration of dental implants coated with collagen, chondroitin sulfate, and growth factor, showed the positive effect of combination between collagen and chondroitin sulfate [8]. Other experiments on implants with collagen–chondroitin sulfate modified surface have been made to improve osseointegration. The results obtained from preclinical studies using animal models allowed clinical phase I studies on humans, offering a clinical perspective [16].

So far, the recent studies have demonstrated that scaffolds based on collagen and chondroitin sulphate have good biocompatibility and controllable properties, which allow in vivo degradation rates in a certain period of time, making them valuable for a variety of biomedical applications in regenerative medicine [17]. Another application of collagen–chondroitin sulfate with hyaluronic acid finds place in cartilage tissue engineering as hybrid hydrogel scaffold for cartilage regeneration [18].

COLL-based biodegradable composites, by incorporation of hydroxyapatite powder in COLL-CS in form of gel, with potential application as scaffolds for bone tissue engineering, were also synthesized and studied. In the presence of collagenase, scaffold biodegradation was reduced after UV irradiation. The biocompatibility of COLL-based biomaterials was investigated in a rat osteoblast culture and was promising. The composite materials exhibited improved cell proliferation and adhesion compared to the material containing only COLL [19].

The aim of this research was to develop composites based on collagen, chondroitin sulfate, and sage oil, used for the osseointegration process of dental implants. These natural substances support bone regeneration, reduce inflammation, prevent infection, and improve the healing process, thus leading to a rapid and efficient post-operative recovery. As far as we know, in the specialized literature, the effect of these natural compounds has not been studied for this purpose.

Sage oil is known for its antifungal, anti-inflammatory, antibacterial, antitumor, and antioxidant activities, being used as a therapeutic agent that prevents the infection without affecting the surrounding tissue [20,21,22,23].

The effect of sage extracts on oral health was intensively studied. Due to its anti-inflammatory and antibacterial properties, the sage extract in mouthwash was proved to be effective against gingival inflammation and mouth ulcers [24,25]. It was synthesized and analyzed as a toothpaste with activity against dental cavities [26]. Moreover, it was demonstrated that Salvia officinalis is effective in reducing microbial colonization on the surface of the dental implant [27].

Thereby, all the components of the new synthesized composite contribute to the osseointegration of dental implants. COLL is the essential protein in elasticity and tissue resistance, contributing to wound healing. CS is the main component of cartilage and aims to reduce inflammation, and sage oil is well known for its anti-inflammatory and antioxidant properties. The obtained composites based on collagen, chondroitin sulphate and sage essential oils can be used in different fields of application such as wound dressings for wound management and plastic surgery, fillers for bone regeneration in orthopedics and dentistry.

## 2. Materials and Methods

### 2.1. Materials

The botanical material utilized in this study was sourced from the Medicinal and Aromatic Plants collection garden at Hatay Mustafa Kemal University. Following harvest, the leaves of Salvia officinalis were subjected to extraction by water distillation utilizing a Neo-Clevenger apparatus for 2 h.

The type I collagen gel of bovine origin with an initial concentration of 2.42% (dry substance) and acidic pH (2–3) was prepared following the known technology previously described [28]. Briefly, the bovine dermis was swollen in organic acid and then the fat was mechanically removed; to remove the non-collagenous protein, the obtained dermis was thus washed and treated with alkali and then washed until neutralization. Then the obtained gel was solubilized again in acid, following several times precipitation and resolubilization until a viscous type I collagen gel was obtained.

Chondroitin sulfate (CS) was purchased from Sigma (Steinheim, Germany), sodium hydroxide (NaOH), and glutaraldehyde (GA) were from Merck (Burlington, MA, USA).

### 2.2. Gas Chromatography-Mass Spectrometry (GC-MS) Analysis of Sage Oil

The volatile chemical profile of the essential oil samples was determined by Gas Chromatography-Mass Spectrometry (GC-MS) using a Thermo Scientific Focus system. Chromatographic separation was performed on a TR-5MS capillary column (60 m × 0.25 mm i.d., 0.25 µm film thickness) with a constant helium carrier gas flow of 1.0 mL/min. The mass spectrometer was operated in electron impact mode at 70 eV, collecting data in full scan mode across a mass range of *m*/*z* 50–650. Critical temperature settings were maintained as follows: the injection port at 220 °C, the ion source at 220 °C, and the MS transfer line at 250 °C. Sample introduction involved a 1 µL injection performed in split mode with a ratio of 250:1. The oven temperature was programmed to increase from an initial 50 °C to a final 220 °C at a steady rate of 3 °C per minute. The constituent identification was achieved by cross-referencing the acquired mass spectra with the Wiley mass spectral library. Furthermore, retention indices for all volatile compounds were determined by calibration with a homologous series of n-alkane standards (C8–C20 and C21–C40). All data acquisition and processing were conducted using the Xcalibur 2.1 software suite.

### 2.3. Preparation of Spongious Form

The collagen gel having a 1% concentration in collagen was mixed with 0.3 or 0.6% chondroitin sulfate (CS) and 0.001 or 0.002% sage essential oils (SEO) as is indicated in Table 1. The collagen gels with CS and SEO were adjusted to physiological pH (7.2–7.4) using NaOH (0.1 M). The obtained gels (Coll, CSS1 ÷ CSS6) were crosslinked with GA, cast in glass Petri dishes for 24 h at 4 °C and then placed on the previously cooled lyophylizer shelves (at −40 °C) of the freeze-dryer (Martin Christ LSC Delta 2-24 freeze-dryer, Osterode am Harz, Germany).

The compositions and codes of gels are presented in Table 1.

The shelves were maintained at the same temperature (−40 °C) for 6 h without pressure. Then, the process continued with main freeze-drying at the same temperature, but at 0.12 mbar for another 8 h. After this, the temperature was increased to 10 °C in 10 h, then to 20 °C for 10 h, and then to 30 °C in 10 h at the same pressure. The samples were dried in the end for 2 h at 30 °C and 0.001 mbar and 2 h at 35 °C and 0.001 mbar as final freeze-drying. Thus, the spongious forms of gels from Table 1 were obtained and characterized by stability in collagenase solution, by electronical microscopy, water absorption and by structural behavior with FT-IR. The biocompatibility was tested on composites with human bone marrow-derived mesenchymal stem cells (BMSCs) and human osteosarcoma cell line MG63.

### 2.4. Water Uptake of Spongious Forms

The pieces with about 1 cm^3^ spongious forms (freeze-dried gels) were used to determine the water uptake capacity, using the method as we previously described [29]. Briefly, the samples of spongious forms were weighed at room temperature (about 20 °C), before and after immersion in water at different intervals of time (after one hour, 12, 14, 48, and 72 h). The following equation (Equation (1)) was used to determine the water uptake:

*Water uptake* (%) = [(*Wt* − *Wd*)/*Wd*] × 100 (1)

where Wt was the weight of water kept by sponges at time t, and Wd was the weight of dry sponges. The data were presented as mean ± standard deviation (SD) of three independent experiments.

### 2.5. The Biodegradation of Spongious Forms

The stability of spongious biocomposites was performed by in vitro degradation in collagenase solution (10^−6^ mg/mL) in phosphate saline buffer (PBS) at 7.4 pH at 37 °C. The sponges were kept in water for 24 h to reach an equilibrium and then were placed in collagenase solution (3 mL). At specific time intervals (first at 2, 4, 6, and 24 h and then about every day, until day 11), the sponges were weighed, and the weight loss was calculated using the following equation (Equation (2)):

*Weight loss* (%) = [(*Wo* − *Wt*)/*Wo*] × 100 (2)

where Wo was the weight of sponges after saturation with water, and Wt was the weight of sponges after the immersion in collagenase solution at time t. This experiment was performed in triplicate.

### 2.6. Fourier-Transform Infrared Spectroscopy (FTIR) of Spongious Forms

The spectral evaluation of the spongious forms were characterized using a Jasco FT/IR-4X spectrometer fitted with an ATR PRO ONE accessory (Jasco, Tokyo, Japan). All spectra were recorded in the wavenumber range between 4000 and 500 cm^−1^ at room temperature and 4 cm^−1^ as nominal spectral resolution. Each final spectrum represents the average of 64 scans per sample.

### 2.7. Scanning Electron Microscopy (SEM) of Spongious Forms

The spongious forms present a porous morphology which was observed using the TM4000 Plus tabletop scanning electron microscope (Hitachi, Tokyo, Japan). The samples were analyzed without being covered with a conductive layer, using a voltage of 15 kV and a magnification of 100×.

### 2.8. Assessment of Biocompatibility

To test the biocompatibility of the collagen scaffolds, we used MG63 (CRL-1427, ATCC), a human osteosarcoma cell line suitable for assessing interaction with biomaterials designed for bone repair. Thus, we first tested the cytotoxicity and the capacity to support viability of extracts obtained by incubating the scaffolds in culture medium, followed by assessment of the scaffold’s capacity to sustain colonization with MG63 cells.

The tested scaffolds were incubated in culture medium (low glucose Dulbecco’s Modified Eagle Medium from Sigma Aldrich, St. Louis, MO, USA, supplemented with 10% (*v*/*v*) fetal bovine serum from Gibco BRL, Gaithersburg, MD, USA, and 100 IU/mL penicillin, 100 µg/mL streptomycin, 50 µg/mL neomycin, all from Sigma Aldrich, St. Louis, MO, USA), at a concentration of 2 mg/mL, for 6 h/37 °C, under continuous stirring. Next, the samples were centrifuged for 5 min at 300× *g* and the supernatants were sterilized by filtration (0.2 μm pore size).

### 2.9. Cytotoxicity and Viability Assays

Cells were seeded at a density of 10^4^/cm^2^ in 96-well plates. Twenty-four hours after seeding, the extracts were added in triplicate and incubated for an additional 24 h for cytotoxicity assessment (LDH assay) and up to 72 h for viability evaluation (XTT assay). LDH assay was performed using the Cytotoxicity Detection Kit (Roche, Basel, Switzerland) following the manufacturer’s instructions. The positive control (high control) for the LDH release was obtained by adding 5 µL/well of Lysis buffer onto the cells 15 min before the test. The negative control was represented by cells cultured in complete growth medium with COLL extract. Lactate dehydrogenase activity was determined by adding 100 µL of freshly prepared reaction mix to each well and incubating for 30 min protected from light. Next, 50 µL of stop solution was added, and the absorbance was measured at 490 nm versus 600 nm, using a TECAN spectrophotometer (Männedorf, Switzerland). The results were expressed as percentages of the High Control.

The viability was evaluated in triplicates by XTT assay (Thermo Fisher Scientific, Waltham, MA, USA), 24, and 72 h after the addition of the extracts. Briefly, cells were washed with PBS and incubated with 100 µL/well XTT working solution following the manufacturer’s instructions, for 2 h at 37 °C, 5% CO_2_. The absorbance was read at 450 nm versus 690 nm using a TECAN spectrophotometer. The results were expressed as a percentage of the COLL control.

Furthermore, the capacity of the collagen scaffolds to support *colonization with MG63 cells* was assessed. For this purpose, scaffold pieces were cropped with a 4 mm-diameter punch. The materials were sterilized by incubation in 70% ethanol overnight under stirring, washed with sterile water, and maintained in DMEM without serum for at least 24 h. Afterwards, 5 × 10^4^ cells were seeded onto the cropped scaffolds and cultured up to 6 weeks. After 2, respectively, 6 weeks, the samples were fixed in 4% PFA and processed for paraffin embedding. By using a Leica microtome, 5 µm thick slices were obtained, which were later subjected to haematoxylin-eosin and Gomoris’s Trichrome staining. For haematoxylin-eosin staining, the slices were incubated for 1 min in haematoxylin and 30 s in eosin Y. The Trichrome Masson staining was performed according to the manufacturer’s instructions (Thermo Fisher Scientific, Waltham, MA, USA). Subsequently, the samples were mounted in Shandon Consul-Mount (Thermo Fisher Scientific, Waltham, MA, USA), and visualized using a Zeiss Observer D1 microscope.

Data for cytotoxicity and viability assays were analyzed using One-way ANOVA and are presented as mean ± standard deviation (SD) from three independent experiments, measured in triplicate. (*** *p*< 0.001; **** *p* < 0.0001).

## 3. Results and Discussion

**Figure 1:**
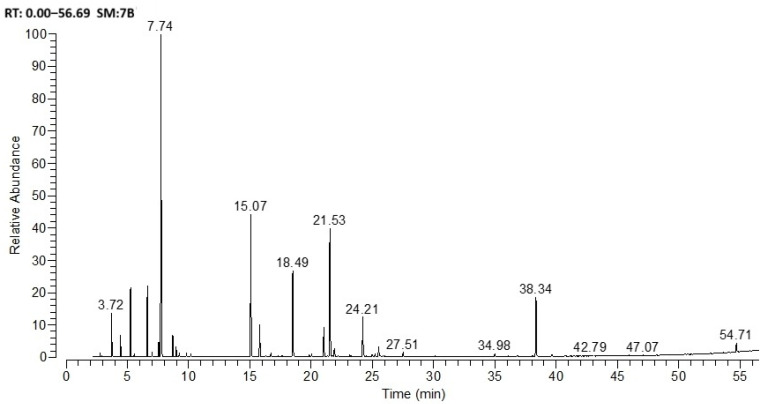
GC–MS chromatogram of *Salvia officinalis* L.

Figure 1 and Table 2 report the composition of *Salvia officinalis* essential oil samples, determined by GC-MS.

The major components of the *Salvia officinalis* L. (sage) essential oil used in the study were identified as eucalyptol (24.84%), camphor (18.71%), thujone (16.02%), and trans-caryophyllene (12.44%).

The obtained oil of salvia was embedded in samples CS1, CS2, CS6, and CS7 in proportions of 0.001 and 0.002% as shown in Table 1. The sage essential oil demonstrated a reaction between its components and collagen and chondroitin sulfate, altering the water absorption and enzymatic degradation properties.

**Figure 2:**
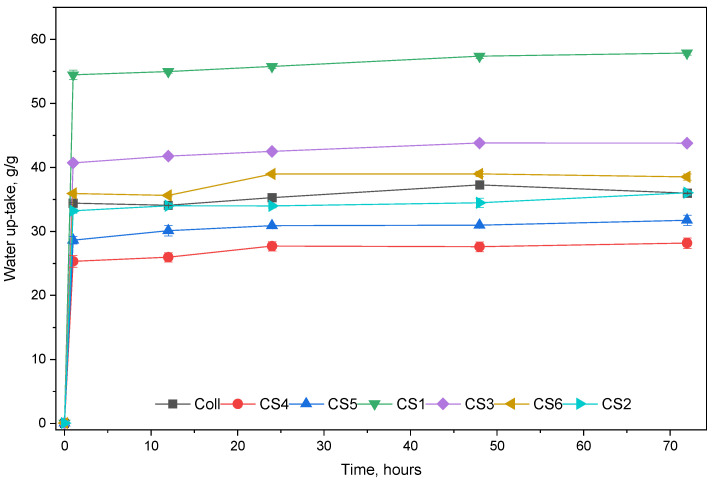
Water up-take of spongious forms Coll and CS1–CS6.

Figure 2 presents the water uptake for the spongious forms. The samples containing only collagen and sage EO absorbed less water than Coll, with a maximum of 28.16 g/g CS1 and 31.73 g/g CS2, compared with 35.96 g/g Coll. This may be due to hydrophobic properties of components of sage EO, such as camphor, eucalyptol (which is insoluble in water) thujone and trans-caryophyllene, which are the main components of sage EO. On the contrary, the water uptake improved with increasing amounts of chondroitin sulfate, which has a hydrophilic nature. The samples CS3 and CS4 absorbed up to 57.86 g/g and 43.77 g/g, respectively. The interesting behavior takes place when the spongious forms combine all the components, collagen, chondroitin sulfate, and sage EO. The results of water uptake for CS 5 and CS 6 are about 38.49 g/g and 36.01 g/g.

**Figure 3:**
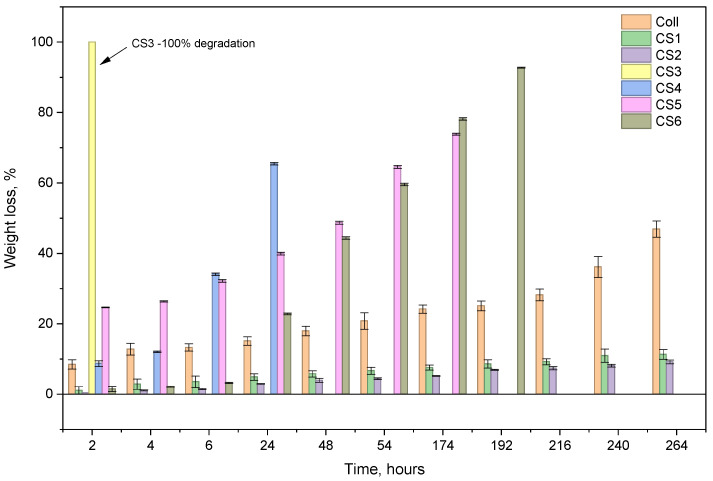
Enzymatic degradation of spongious forms Coll and CS1–CS6.

The results of water absorption showed the influence of sage EO and chondroitin sulfate on collagen properties. All the samples absorbed the most water in the first hour and reached equilibrium in 24 h; thereafter, the amount of absorbed water decreased significantly. Figure 3 presents the enzymatic degradation in collagenase solution for the studied samples for 11 days.

The most resistant samples are the ones with EO sage oil, due to crosslinking of their components (aldehyde or ketone) with collagen and, probably, to their inhibitor effect on collagenase. On the contrary, the sample with the highest amount of chondroitin sulfate degrades very fast; CS3 resisted only one hour in collagenase solution. The samples with sage oil degrade by small percentages: 11.27% for CS1 and 9.17% for CS2. The samples that contain both essential oils and chondroitin sulfate, CS 5 and CS6, degrade totally after 9 and 8 days, respectively. The results corelate with those of water absorption.

**Figure 4:**
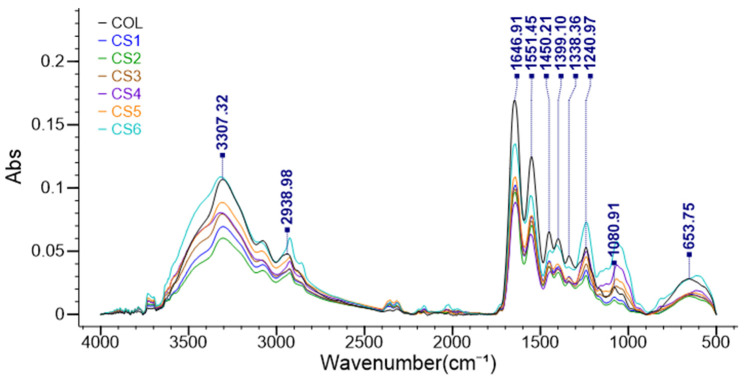
FT-IR spectra of spongious forms Coll and CS1-CS6.

The physical–chemical interactions between components of the spongious forms were highlighted by FT-IR spectroscopy. Figure 4 presents the FT-IR spectra of all the spongious forms.

It is well known that the typical FTIR collagen spectra consist of Amide I (about 1650 cm^−1^), Amide II (1550 cm^−1^), Amide III (1240 cm^−1^), Amide A (3300 cm^−1^), and Amide B (2930 cm^−1^) bands. The characteristic peaks in chondroitin sulfate’s FTIR spectrum are between 1606 and 1651 cm^−1^ (amide group), 1223–1231 cm^−1^ (peaks for the sulfate group), and 829–854 cm^−1^ (indicating the presence of a bond in the glycosidic linkage). Comparing the Coll spectrum with the CS1÷CS6 ones, it can be noticed that the collagen-specific structure is not affected by essential oils or chondroitin sulphate. The peak from 1080 cm^−1^ from Coll was shifted to 1066–1070 cm^−1^ when chondroitin sulphate was present in samples.

**Figure 5:**
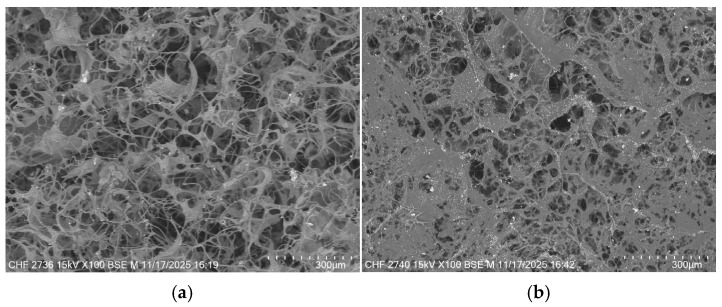

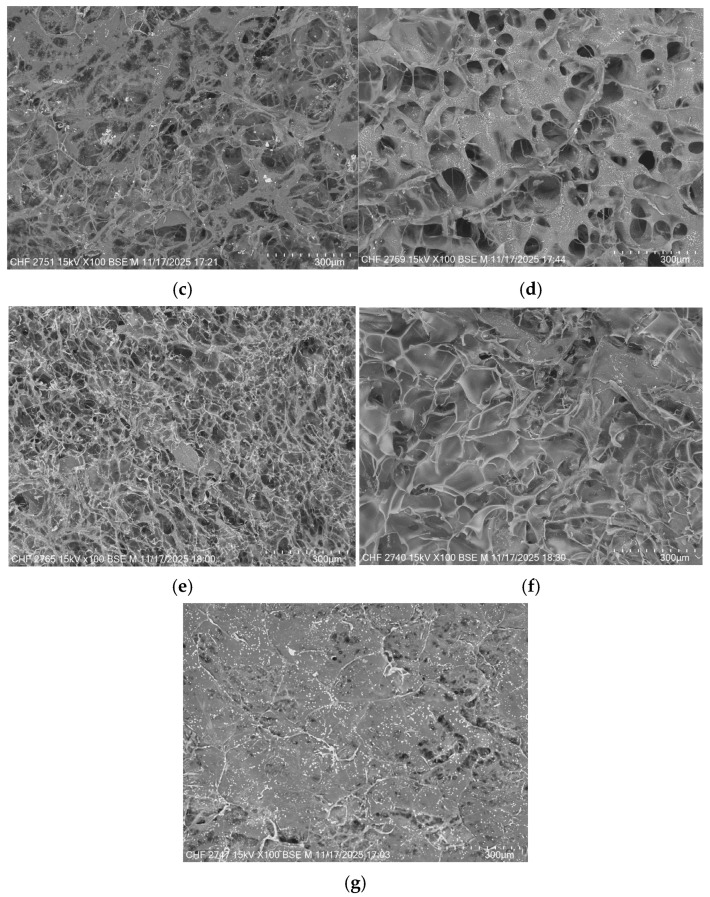
SEM images of spongious forms: (**a**) Coll; (**b**) CS1; (**c**) CS2; (**d**) CS3; (**e**) CS4; (**f**) CS5 and (**g**) CS6 (magnification ×100).

The morphological structure of collagen was also proved by the SEM images, which showed for all the samples a structure with interconnected pores, as is presented in Figure 5.

As Figure 5 shows, both CS and Sage EO influence the morphological structure of collagen. The control sample Coll presents a uniform structure with pore sizes of about 100–200 µm. The samples with sage EO exhibit more compact structures with smaller pores at higher EO content. The chondroitin sulphate leads to more uniform structures with smaller pore sizes than the control sample Coll. The results are in correlation with water absorption and enzymatic degradation and showed that an interconnected structure can be tuned by varying the amount of CS or sage oil.

**Figure 6:**
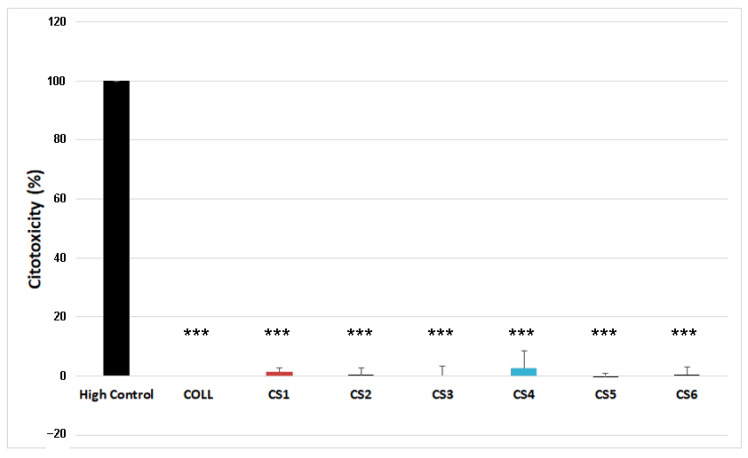
Evaluation of the cytotoxic effect of the collagen extracts on MG63 cells after 24 h exposure evaluated by LDH assay. Lysed cells were used as positive (high) control. Results are given as mean ± standard deviation of 3 independent experiments (*n* = 3, *** *p* < 0.001 versus High control). There was no statistical significance between CS1–CS6 samples and COLL control or among each other.

As shown in Figure 6, none of the tested scaffold extracts showed cytotoxic activity.

**Figure 7:**
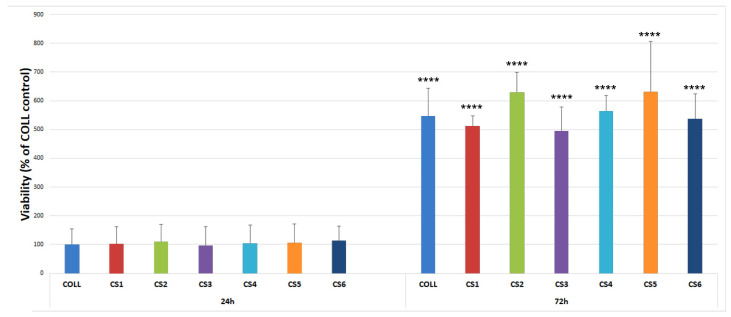
Viability of MG63 cells incubated in the presence of the extracts determined by XTT assay performed after 24, and 72 h. The data is presented as mean ± standard deviation of 3 independent experiments (**** *p* < 0.0001 versus each counterpart at 24 h). There was no statistical significance between samples at each checkpoint.

Furthermore, the results shown in Figure 7 indicated that all tested extracts supported MG63 viability similarly. No statistical significance was obtained between the CS1-CS6 samples or versus COLL control at 24 h or 48 h checkpoints.

**Figure 8:**
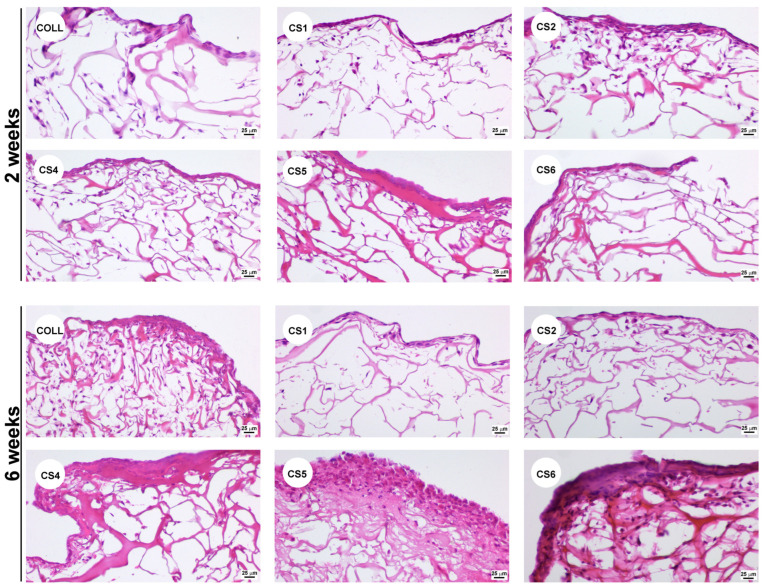
Haematoxylin and Eosin staining illustrating the colonization of collagen scaffolds with MG63 cells.

Next, we assessed the capacity of the tested scaffolds to sustain the growth of MG63 cells. The Haematoxylin and Eosin histological staining showed that all samples sustained the three-dimensional growth of MG63 osteoblasts, not only at the surface but also within the scaffolds, distributed throughout the structural porosity (Figure 8).

**Figure 9:**
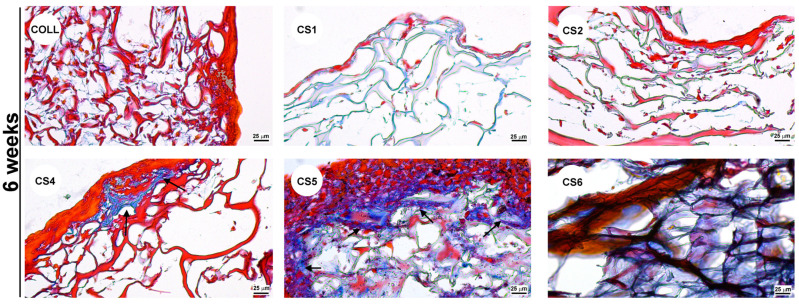
Gomoris’s Trichrome staining showing the collagen deposition by MG63 cells on collagen scaffolds. Collagen deposits (blue) are indicated with black arrows.

In the case of CS3, the scaffold was completely degraded after 24 h when incubated in complete culture medium; therefore, it could not be tested for its ability to support colonization. All the other scaffolds maintained structural integrity up to 6 weeks in culture. As shown in Figure 8, after 2 weeks, all samples were able to support cell growth similarly to the COLL control. On the contrary, long-term culture indicated that after 6 weeks, cells were not abundant in the CS1 and CS2 samples, compared to the COLL control. In contrast, CS4, CS5, and CS6 samples provided not only a good environment for cell growth, but also promoted the deposition of abundant extracellular matrix, corroborating the presence of chondroitin sulfate in these samples. To further emphasize the matrix deposition, Gomoris’s Trichrome staining was performed. As noticed in Figure 9, the newly formed collagen was stained in blue, while the collagen sponge was stained mainly in red color, which highlighted the collagen deposited by the cells during the 6 weeks in culture.

It can be noticed that collagen deposits can be observed predominantly in samples CS4, CS5, and CS6, indicating that the extracellular matrix secreted by MG63 cells was composed mostly of collagen. However, no calcification was observed under the culture conditions we used. We can safely assume that to promote the calcification process, specific differentiation conditions should be used.

The enhanced cell colonization and extracellular matrix deposition observed in samples CS4-CS6 could be attributed to the presence of chondroitin sulfate, which is known to have a pivotal role in osteogenic differentiation [30]. Previous studies have shown that integrating chondroitin sulfate integration in various biomaterials can have a beneficial impact for bone regeneration [31]. In addition, the presence of sage oil within these samples may further contribute through its anti-inflammatory and antioxidant properties [32], creating a more favorable microenvironment for cell growth and tissue integration. Thus, controlled incorporation of chondroitin sulfate and sage oil into collagen scaffolds appears to be a promising approach for enhancing bone tissue regeneration.

## 4. Conclusions

This research aimed to develop new sponge forms as a regenerative scaffold for bone tissue engineering. The sponge forms consist of collagen, chondroitin sulfate, and sage essential oils. The samples were characterized by GC-MS for essential oils, water uptake, biodegradation analysis, FT-IR, and scanning electron microscopy, and biocompatibility with the MG-63 human osteosarcoma cell line. The results showed that the samples with sage essential oils are most resistant, and the ones with chondroitin sulfate promoted the deposition of abundant extracellular matrix. Thus, controlled incorporation of chondroitin sulfate and sage oil into collagen scaffolds is likely to strengthen their potential in bone engineering applications.

## Figures and Tables

**Table 1:** Composition of collagen gels.

Codes of Samples	Collagen *, %	Chondroitin Sulfate **, %	Sage oil *, %	GA **, %
Coll	1	0	0	0.25
CS 1	1	0	0.001	0.25
CS 2	1	0	0.002	0.25
CS 3	1	0.6	0	0.25
CS 4	1	0.3	0	0.25
CS 5	1	0.6	0.001	0.25
CS 6	1	0.3	0.002	0.25

* Reported to gel amount as volume. ** Reported to collagen, dried substance as mass.

**Table 2:** Components of *Salvia officinalis* L. essential oil.

RT	RI	Components	% *
3.72	1027	α–Pinene	2.40
4.35	1072	Camphene	1.32
5.13	1111	β–Pinene	4.53
6.48	1169	α–Myrcene	4.71
7.40	1200	dl–Limonene	1.09
7.53	1205	Bornylene	1.00
7.74	1207	Eucalyptol	24.84
8.70	1246	cis–Ocimene	1.60
15.07	1421	Thujone	16.02
18.49	1508	Camphor	18.71
20.89	1576	Endobornyl acetate	2.84
21.53	1587	trans–Caryophyllene	12.44
24.21	1660	α–Humulene	3.84
25.42	1696	α–Terpineol	1.02
34.81	1967	Caryophyllene oxide	1.00
38.34	2074	Veridiflorol	6.10
54.50	2647	Sclareol	1.00

* The table lists components with a concentration of 1% or higher.

## Data Availability

Data is contained within the article.
